# The effect of diabetes mellitus on oral health-related quality of life: A systematic review and meta-analysis study

**DOI:** 10.3389/fpubh.2023.1112008

**Published:** 2023-02-24

**Authors:** Yousef Mohseni Homagarani, Kimia Adlparvar, Saba Teimuri, Mohammad Javad Tarrahi, Firoozeh Nilchian

**Affiliations:** ^1^Student Research Committee, School of Dentistry, Isfahan University of Medical Sciences, Isfahan, Iran; ^2^Department of Epidemiology and Biostatistics, School of Health, Isfahan University of Medical Sciences, Isfahan, Iran; ^3^Dental Research Center, Dental Research Institute, Isfahan University of Medical Sciences, Isfahan, Iran

**Keywords:** diabetes mellitus, quality of life, oral health, oral health-related quality of life, systematic review

## Abstract

**Background:**

Diabetes mellitus is related to variable dental and oral complications like xerostomia and periodontal problems. Therefore, diabetes can affect the oral status and oral health-related quality of life (OHRQoL). A systematic review of evidence can determine the association between diabetes and OHRQoL. so, this study aimed to evaluate the effects of diabetes on OHRQoL.

**Methods:**

After determining the PECO and eligible criteria, a comprehensive search was conducted in PubMed, Scopus, Web of Science, and Embase without any restriction. Further searches were performed in Google Scholar and reference lists of selected articles. Two independent reviewers carried out paper selection, data extraction, and quality assessment. A meta-analysis was conducted using a “random effect model” and the standardized mean difference of OHRQoL with a 95% confidence interval (CI) was reported as estimating pooled effect size.

**Results:**

After screening 237 identified records, three case-control and ten cross-sectional studies met eligibility criteria. Two cross-sectional studies were excluded in the quality assessment phase and the rest of the studies have a low or moderate risk of bias. The pooled standardized mean difference between the case and the control groups was 0.148 (95% CI: −0.045 to 0.340).

**Conclusion:**

Diabetes mellitus has no statistical significant association with OHRQoL. Nevertheless, based on the articles' review, it seems that diabetes can lead to functional limitations, physical pain, and psychological discomfort. Also, complications of diabetes adversely affect wellbeing. Hence dentists can play an essential role in the awareness of persons with diabetes about these problems and improve their OHRQoL.

**Systematic review registration:**

https://www.crd.york.ac.uk/prospero/display_record.php?ID=CRD42022303038, identifier CRD42022303038.

## 1. Introduction

Diabetes mellitus is a chronic disease caused by insulin dysfunction and deficiency characterized by hyperglycemia (high blood glucose) (1, 2). Chronic hyperglycemia causes defects and failure in different body parts like the nervous system, eyes, kidneys, and cardiovascular system (3–5).

International Diabetes Federation (IDF) estimated that in 2021, 536.6 million adults had diabetes in 215 countries. It also stated that by 2,045, approximately 783.2 million people worldwide will have diabetes mellitus (6).

Diabetes also relates to variable dental and oral complications, consisting of dental caries (7), gingival problems, periodontal abscess, and periodontitis (8, 9), xerostomia (7), vesiculobullous lesions, oral fungal infections, increased possibility of post-operative infections, and impaired wound healing (10–12).

Several studies consider that oral health complications can adversely affect physical function, social status, psychological comfort, and emotional state (13–15). Therefore, the relationship between oral and dental health and its possible consequences on quality of life is undeniable. Additionally, some original studies considered diabetes as a factor that may affect oral health-related quality of life (OHRQoL) (16–20). On that account, OHRQoL attracts dental practitioners' and researchers' attention toward itself.

OHRQoL is one of the subsets of health-related quality of life that examines the effect of oral health and status on quality of life. OHRQoL reflects people's comfort when eating, sleeping, and engaging in social interaction; their self-esteem; and their satisfaction concerning their oral health (21–23). OHRQoL is mainly assessed by questionnaires such as Oral Health Impact Profile (OHIP) and Geriatric Oral Health Assessment Index (GOHAI). OHIP is The most widely available questionnaire for quantification of OHRQoL, which measures the seven domains of functional limitation, physical pain, psychological discomfort, physical disability, psychological disability, social disability, and handicap (24).

Thus, our study aimed to perform a systematic review and meta-analysis to evaluate the effects of diabetes on oral health-related quality of life.

## 2. Methods

### 2.1. Protocol and registry

This systematic review and meta-analysis were carried out following the principles of the Preferred Reporting Items for Systematic Reviews and Meta-Analyses (25), and the protocol was registered to PROSPERO (reg. no. CRD42022303038).

### 2.2. PECO question

Does diabetes mellitus affect oral health-related quality of life in diabetic adults?

### 2.3. Eligibility criteria

Observational studies (cross-sectional, case-control, and cohort) that examined diabetes mellitus as the exposure and OHRQoL as the outcome (determined by OHIP or GOHAI questionnaire) and also studied individuals over 18 years of age were included. Studies in which participants had received periodontal treatment in the past 6 months were excluded, along with case reports, reviews, and review protocols. In addition, no restrictions on language or year of publication were considered.

### 2.4. Literature search and screening

Two independent reviewers (YMH and KA) carried out a systematic literature search using PubMed, ISI Web of Science, EMBASE, and Scopus without any restriction on language or other limitations until April 2022. Further searches were conducted in Google Scholar (gray literature) and reference lists of selected articles until May 2022. We used following search query in PubMed: (“diabetic patient^*^”[All Fields] OR “diabet^*^”[All Fields] OR “Diabetes Mellitus”[MeSH Terms] OR “Diabetes Mellitus”[All Fields] OR “diabetes mellitus, type 1”[MeSH Terms] OR “diabetes mellitus type 1”[All Fields] OR “diabetes mellitus, type 2”[MeSH Terms] OR “diabetes mellitus type 2”[All Fields] OR “T1DM”[All Fields] OR “T2DM”[All Fields]) AND (“Oral Health Related Quality of Life”[All Fields] OR “OHRQoL”[All Fields] OR “OHRQL”[All Fields]). The search strategies of other databases are also presented in Supplementary Table 1.

After removing duplicated studies, two independent reviewers (KM and ST) screened the title and abstract of studies according to eligibility criteria. Dubious studies were kept for the next stage. Then, the remaining full-text articles were evaluated according to inclusion and exclusion criteria, and relevant reports were selected. Any disagreements about the selection of articles between two reviewers were resolved by consulting the third reviewer (YMH).

### 2.5. Quality assessment

The same two reviewers (KM and ST) independently evaluated the quality of studies using NIH quality assessment tools for Observational Cohort and Cross-Sectional Studies and Case-Control Studies (26). For the former, scores of 0–4 were considered poor, 5–9 were considered fair, and scores of 10–14 were considered good quality. For the latter, scores of 0–4 (poor), 5–8 (fair), and 9–12 (good) were considered to assess the quality of the included studies.

### 2.6. Data extraction

A data extraction spreadsheet was designed, and the following information was independently extracted by two reviewers (KM and ST): first author's name, year of publication, country, study design (Cross-Sectional, Case-Control, and Cohort studies), participant characteristics (sample size, gender, and age), type of questionnaire and statistical summaries related to OHRQoL (effect measures, confidence intervals, and *p*-values).

### 2.7. Statistical methods and data synthesis

The meta-analysis was conducted using Comprehensive Meta-Analysis software (V3.7). The standardized mean difference (SMD) of OHRQoL with a 95% confidence interval (CI) was reported as estimating pooled effect size. Due to the small number of studies, a “random effect model” was used. Heterogeneity across included studies assessed by the Cochran Q test and I-square index. If the *I*^2^ statistic is greater than 50%, heterogeneity may represent Moderate to substantial (27). Publication bias was determined by funnel plot based on Begg's test. In the funnel plot, SMD against standard error was presented. The symmetric funnel plot shows lower biases in results.

Due to the lack of a clear cutoff to determine the effect of OHRQoL on different variables such as diabetes, only the standardized mean difference (SMD) of OHRQoL between the case and control groups was used for meta-analysis.

### 2.8. Ethics approval

This research was approved by the Research Ethics Committees of Vice-Chancellor in Research Affairs -Medical University of Isfahan (Approval ID: IR.MUI.RESEARCH.REC.1400.421).

## 3. Results

### 3.1. Search and study selection

**Figure 1:**
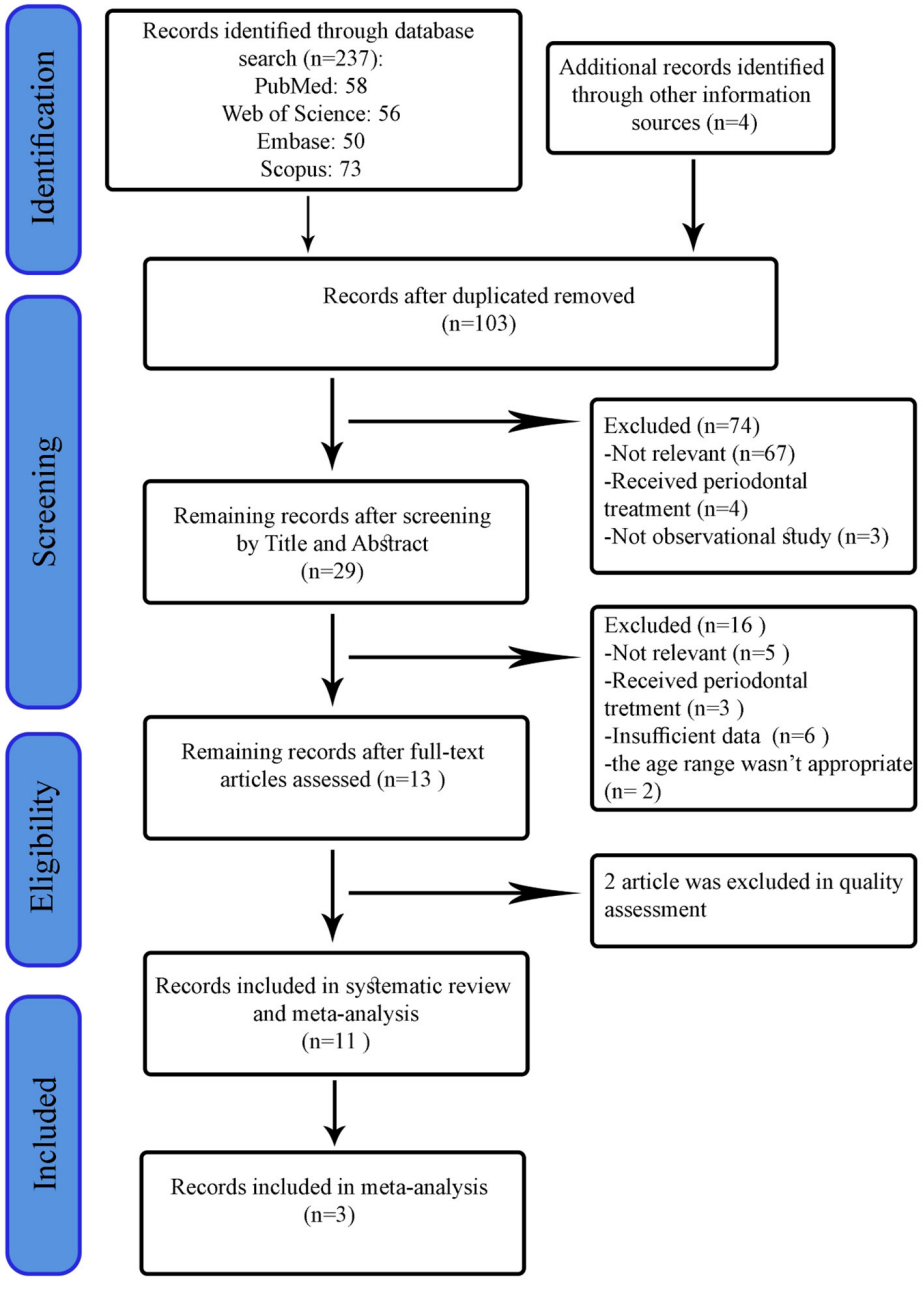
PRISMA flow diagram of search strategy and outcomes.

Initially, 237 records were identified from searching in electronic databases and manual search. After removing duplicates, the title and abstract of 103 articles were screened separately by two reviewers. Based on predetermined eligibility criteria, 29 articles were selected for the full-text stage. After scanning the full-text of articles, 16 records were excluded for the following reasons: the outcome wasn't OHRQoL; the age range wasn't appropriate; Insufficient data to investigate the relationship between diabetes and OHRQoL, and periodontal treatments. In addition, two records were excluded during the quality assessment phase (28, 29). Finally, we used eight articles for qualitative review and three articles for quantitative synthesis. The process of study selection flow diagram is shown in Figure 1.

### 3.2. Study characteristics

Characteristics of included studies are displayed in Table 1. eight of eleven studies employed a cross-sectional design, and the remaining three were case-control studies. Included studies used OHIP 14 (*n* = 7), OHIP 20 (*n* = 3), GOHAI (*n* = 2), and both the OHIP 14 and GOHAI (*n* = 1) for measuring the OHRQoL. The sample size varied between included studies, and the age of the diabetic patients ranged from 18 to 86 years old. Three studies included controlled diabetic patients with type 1 and 2 diabetes [62, 66, and 80% of the participants in Azogui-Lévy et al. (17), Sadeghi et al. (20), and Allen et al. (35) studies had type 2 diabetes, respectively], and controlled diabetic patients in other studies had only type 2 diabetes.

**Table 1:** Characteristics of included studies.

**References**	**Study design**	**Country**	**Sample size (%men, %women)**	**Mean age ±SD (range), years**	**Type of questionnaire**	**Outcomes**
Ravindranath et al. (18)	Cross-Sectional	India	350 (55.1%, 44.9%)	NR^*^ (30–60)	OHIP20, score 1 to 6 for each question (from “never” to “all the time”)	• Functional limitation showed the highest mean score followed by psychological discomfort and Physical pain, respectively • Partially edentulous patients reported a higher mean score than completely edentulous patients • Lower score (76.48 ± 7.98) in patients with DMFT of 0 compared to patients with DMFT of 1–16 (87.68 ± 5.79) • No statistically significant difference between patients with and without dental caries • Periodontal indicators like bleeding on probing, presence of pocket, and loss of attachment had a significant association with higher OHIP scores • Patients who had oral mucosal lesions reported higher OHIP scores but it's not significant • Patients who wore complete dentures had higher scores compared to patients who wore partial dentures
Mohsin et al. (30)	Cross-Sectional	Pakistan	101 (39%, 61%)	53.3 ± 11.0 (≥30)	OHIP14, score 0 to 4 for each question (from “never” to “very often”)	• Functional limitation, psychological discomfort, and physical pain showed the highest score, respectively • Women scored higher than men in all of the domain's OHIP • Oral complications of diabetes don't have a significant effect on OHRQoL
Sadeghi et al. (20)	Cross-Sectional	Iran	250 (44%, 56%)	55.2 (NR)	OHIP20, score 1 to 5 for each question (from “always” to “never”)	• A significant association between OHRQoL and age, knowledge of the link between diabetes and oral complications, educational level, being referred for dental visits by a physician, frequency of brushing, and length of time diagnosed with diabetes • No Significant association between OHRQoL and gender, smoking habits, the type of diabetes, and frequency of dental visits • Diabetes doesn't adversely affect OHRQoL
Drumond-Santana et al. (14)	Cross-Sectional	Ireland	101 (60%, 40%)	56 (31–79)	OHIP20 A Likert response from “never” to “always”	• Diabetes doesn't affect OHRQoL
Verhulst et al. (31)	Cross-Sectional	Netherlands	640 (NR)	NR (≥30)	OHIP14, score 0 to 4 for each question (from “never” to “very often”)	• Physical pain, psychological discomfort, and functional limitation have the most impact on OHRQoL, respectively • Patients with xerostomia, bad breath, and periodontitis have lower OHRQoL • Diabetes doesn't affect OHRQoL
Nikbin et al. (32)	Cross-Sectional	Iran	350 (24.6%, 75.4%)	55.04 ± 10.76 (22–86)	OHIP14 and GOHAI in which both of them have an Score from 1 to 5 for each question (from “always” to “never”)	• Patients with lower OHRQoL had higher CAL and DMFT • Diabetes doesn't adversely affect OHRQoL • Diabetes affects psychological aspects more than functional aspects
Azogui-Lévy et al. (17)	Cross-Sectional	France	281 (70%, 30%)	57 ± 15.4 (NR)	GOHAI score 1 to 5 for each question (from “always” to “never”)	• Wearing of prostheses and dry mouth associated with low GOHAI score • Poor oral health status in type 2 diabetes mellitus adversely affects OHRQoL • Age is inversely associated with lower OHRQoL
de Sousa et al. (33)	Cross-Sectional	Brazil	302 (28.8%, 71.2%)	63.1 (NR)	OHIP14 score 0 to 4 for each question (from “never” to “very often”)	• Physical pain and discomfort associated with lower OHRQoL • Xerostomia, denture need, and periodontitis independent of socioeconomic status have a negative impact on OHRQoL • No association between OHRQoL and socio-demographic, inadequate oral hygiene, dental visits, dental caries, and edentulism
Nayak et al. (16)	Case-Control	India	Case: 138 (NR) Control: 128 (NR)	Case: 53 ± 10.23 (32–75) Control: 52 ± 10.59 (32–73)	OHIP14 score 0 to 4 for each question (from “never” to “very often”)	• Showed poorer OHRQoL among persons with diabetes • Periodontal problems have a negative impact on OHRQoL
Khalifa et al. (19)	Case-Control	UAE	Case: 88 (36%, 64%) Control: 88 (55%, 45%)	Case: 43 ± 1.5 (NR) Control: 43.1 ± 1.5 (NR)	OHIP14 score 0 to 4 for each question (from “never” to “very often”)	• Diabetes doesn't affect OHRQoL • No difference between persons with diabetes and persons without diabetes in terms of dental decay • No significant association between DMFT and OHRQoL • No significant association between CAL and OHRQoL
Pakize et al. (34)	Case-Control	Iran	Case: 250 (50%, 50%) Control: 250 (50%, 50%)	Case: 69.56 ± 6.27 (≥60) Control: 69.68 ± 6.28 (≥60)	GOHAI A Likert response “never” to “always”	• Diabetes doesn't adversely affect OHRQoL • Higher Fast blood sugar associated with lower OHRQoL • Xerostomia and denture need didn't adversely affect OHRQoL

* Not reported.

### 3.3. Assessment of methodological quality

The results of the quality assessment are shown in Tables 2, 3. Based on NIH quality assessment tools, all three case-control studies had a good methodological quality. Except for two studies, the rest of the cross-sectional studies were of fair quality in terms of methodology. The two independent reviewers agreed concerning all items on the scale.

**Table 2:**
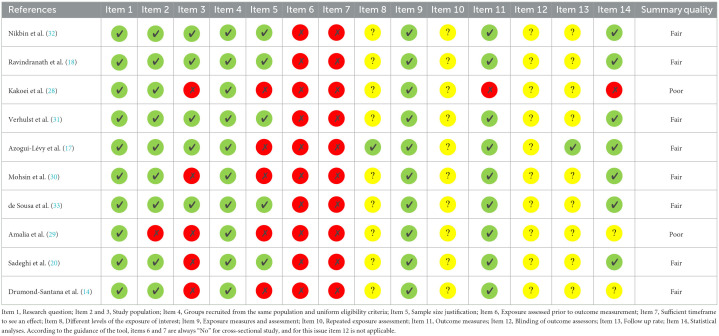
NIH quality assessment tool for observational cohort and cross-sectional studies.

Item 1, Research question; Item 2 and 3, Study population; Item 4, Groups recruited from the same population and uniform eligibility criteria; Item 5, Sample size justification; Item 6, Exposure assessed prior to outcome measurement; Item 7, Sufficient timeframe to see an effect; Item 8, Different levels of the exposure of interest; Item 9, Exposure measures and assessment; Item 10, Repeated exposure assessment; Item 11, Outcome measures; Item 12, Blinding of outcome assessors; Item 13, Follow up rate; Item 14, Statistical analyses. According to the guidance of the tool, items 6 and 7 are always “No” for cross-sectional study, and for this issue item 12 is not applicable.

**Table 3:**
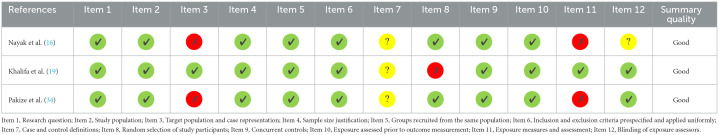
NIH quality assessment tool for case-control studies.

Item 1, Research question; Item 2, Study population; Item 3, Target population and case representation; Item 4, Sample size justification; Item 5, Groups recruited from the same population; Item 6, Inclusion and exclusion criteria prespecified and applied uniformly; Item 7, Case and control definitions; Item 8, Random selection of study participants; Item 9, Concurrent controls; Item 10, Exposure assessed prior to outcome measurement; Item 11, Exposure measures and assessment; Item 12, Blinding of exposure assessors.

### 3.4. Impact on OHRQoL and related variables

Table 1 presents detailed information about the effect of diabetes on OHRQoL and its related variables. Included studies have examined various factors affecting OHRQoL in diabetic patients. Two cross-sectional studies investigated the effect of DMFT on OHRQoL in diabetic patients. Most studies have also examined the effects of periodontal indicators caused by diabetes, such as periodontitis and clinical attachment loss (CAL). One study considered the impact of gender on OHRQoL, while another found no association between gender and OHRQoL. In addition, xerostomia was investigated in most included studies as an effective variable on OHRQoL. Five cross-sectional studies that used the OHIP questionnaire examined different domains affecting OHRQoL in which three domains, Physical pain, psychological discomfort, and functional limitation, were more affected than others.

**Figure 2:**
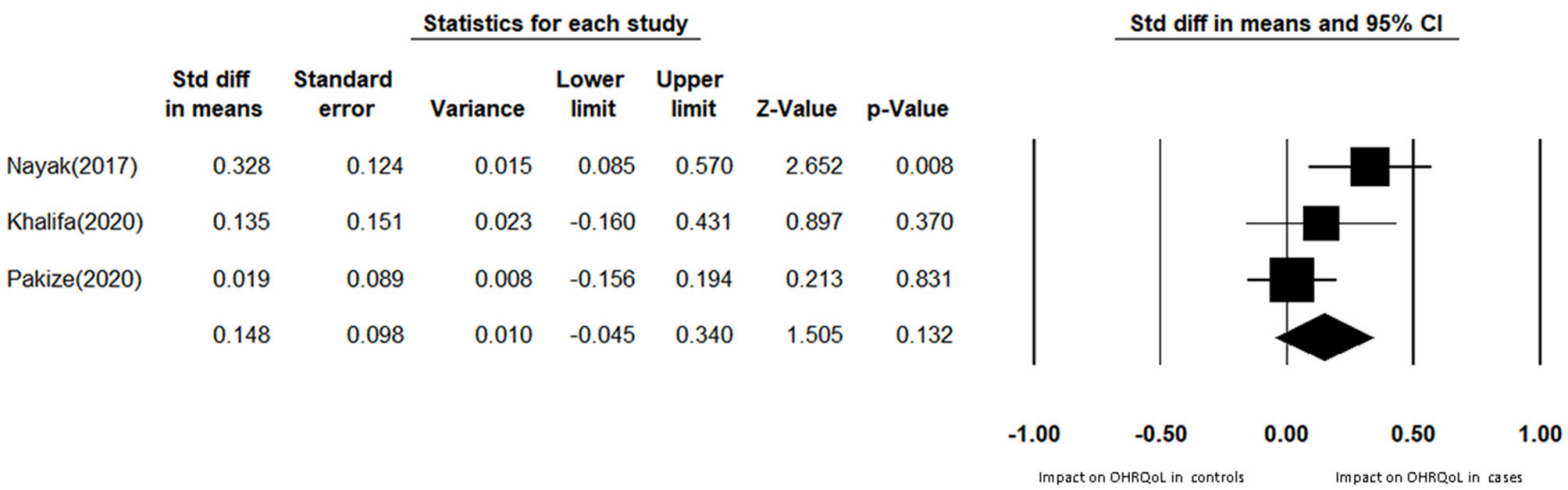
Effect of diabetes on OHRQoL.

**Figure 3:**
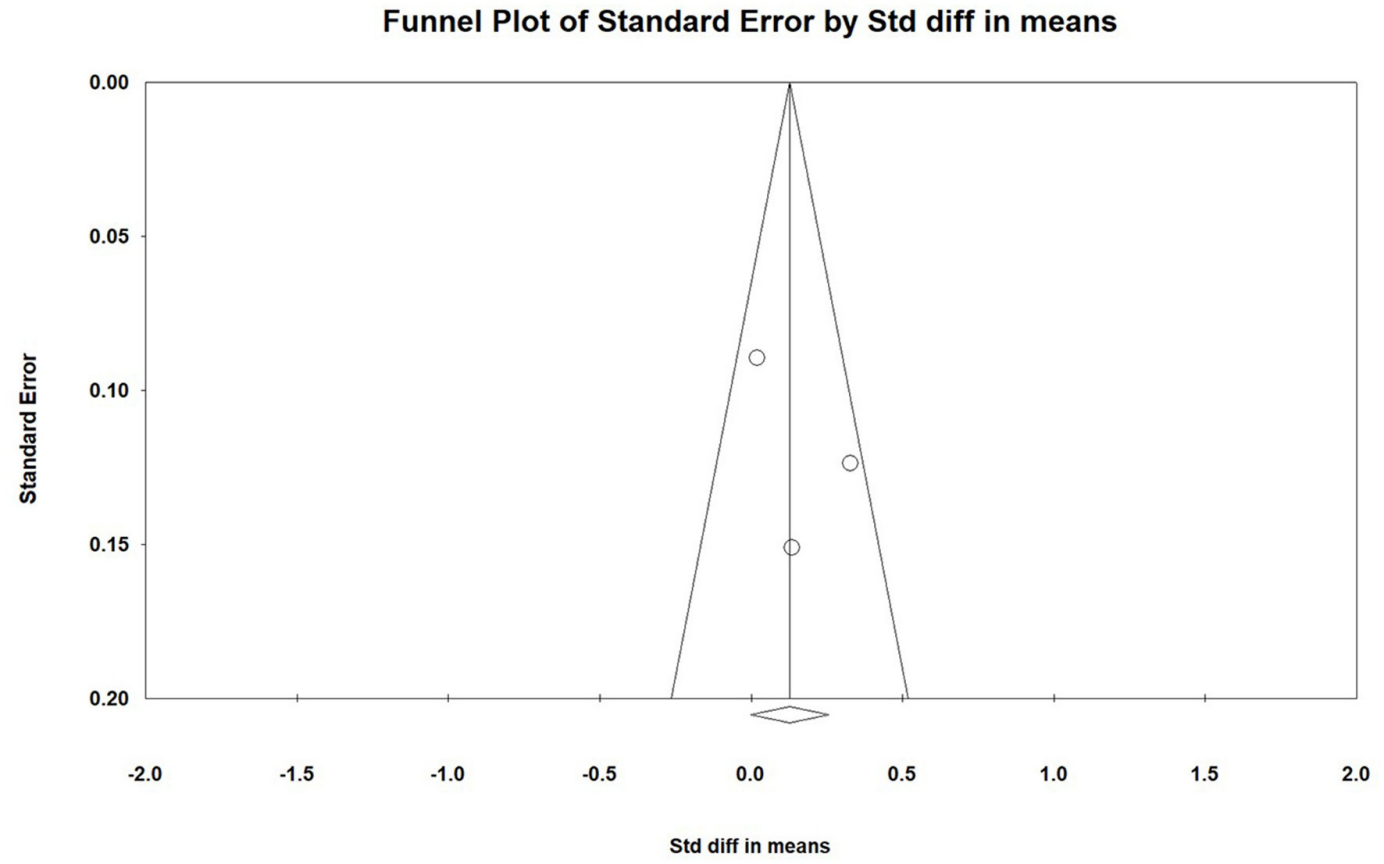
Funnel plot of included studies in meta-analysis.

Due to the lack of a clear universal and academic cutoff point to determine the effect of OHRQoL on different variables such as diabetes, the standardized mean difference of OHRQoL between the case and control groups was used for meta-analysis. Therefore, three existing case-control studies were included in the meta-analysis. The pooled SMD score between case and control groups indicates no impact on OHRQoL among diabetic patients (SMD: 0.148; 95% CI: −0.045 to 0.340; *P* = 0.132; heterogeneity, Cochran Q test = 4.09, *P* = 0.0129 *I*^2^ = 51.16) (Figures 2, 3).

### 3.5. Publication bias

The funnel plot showed a symmetric distribution of the data in each study (Figure 3). Begg's test detected no significant publication bias too.

## 4. Discussion

The present study systematically reviewed the evidence on the impact of diabetes mellitus on oral health-related quality of life and Related variables affecting OHRQoL. Our meta-analysis of three case-control studies (16, 19, 34) revealed no statistically significant association between diabetes mellitus and OHRQoL. Also, in 5 cross-sectional studies, no association was observed between diabetes and OHRQoL (20, 30–32, 35), while three cross-sectional studies indicated the effect of diabetes mellitus on OHRQoL (17, 18, 33). This difference between included studies may be due to the consideration of different cutoffs or their different populations. However, according to the results, it seems that diabetes mellitus has no statistically significant association with OHRQoL. One of the possible reasons for the lack of association between OHRQoL and diabetes is that diabetic patients pay more attention to other aspects of personal health. Therefore, they give more importance to promoting their awareness and general health.

Among the included studies, nine studies used the OHIP questionnaire to assess OHRQoL, of which five studies examined each domain of the OHIP questionnaire separately. All five studies reported a high impact of diabetes on three domains of Functional limitation, psychological discomfort, and physical pain than on other domains. The study of Ravindranath et al. (18) and Mohsin et al. (30) considered the Functional limitation to be more effective than the other two domains, while the other three studies considered the domains of psychological discomfort and physical pain to be more effective (31–33). Therefore, we suggest that physicians and dentists pay more attention to these domains. In other words, they should attend to issue such as diet, chewing problems, pain, sore spots, and concerns related to the oral and dental conditions of diabetic patients in their examinations.

The studies included in this research demonstrated that diabetes mellitus reduced OHRQoL in different ways. Ravindranath et al. (18) and Nikbin et al. (32) showed that higher Decayed, Missing, and Filled Teeth index (DMFT) and clinical attachment loss index (CAL) lead to lower OHRQoL in diabetic patients. However, Khalifa et al. (19) suggested that these two affect OHRQoL regardless of participants' diabetic status. Also, Numerous studies have shown that xerostomia, as one of the most common oral complications of diabetes mellitus reduces the oral health-related quality of life of diabetic patients (31, 33, 34). Like xerostomia, several studies have considered the negative impact of periodontal problems in diabetic patients as a factor in reducing their OHRQoL. Bleeding on probing and the pocket presence have been noticed more than other problems (18, 31, 33).

Mohsen et al. (30) suggested that women's oral health-related quality of life scores were more affected by men, while Sadeghi et al. (20) found no association between gender and OHRQoL scores. Also, based on Sadeghi et al. (20) and Allen et al. (35) study, there is no difference between type 1 and type 2 diabetes concerning OHRQoL. In addition, De Sousa et al. (33) showed no association between OHRQoL and socio-demographic features.

Azogui-Lévy et al. showed that age is directly associated with better OHRQoL. The importance of aesthetic demand in younger patients and the greater adaptation of older people to diabetes after years of suffering from it were among the reasons they stated for this association (17).

Like any research, study limitations must be identified to deduce the finding correctly. Participants in most included studies were gathered from diabetic patients who were referred to hospitals or clinics. In other words, most of the studies were hospital-based. Also, the findings were mainly based on the questionnaire results, and patients were not clinically examined. On the other hand, the small number of case-control studies did not allow for performed meta-analyses with a larger population. Hence, we suggest conducting case-control studies in a large-scale, randomized, and community setting. We also recommend that more dependent variables be carefully examined to determine the confounders and the effect of these variables on OHRQoL.

## 5. Conclusion

Given the finding of this literature, diabetes mellitus has no statistically significant association with oral health-related quality of life. Nevertheless, based on the articles' review, it seems that diabetes can lead to functional limitations as well as physical pain and psychological discomfort. On the other hand, complications of diabetes such as xerostomia and periodontal problems adversely affect wellbeing. Therefore, due to the relationship between some variables related to diabetes and OHRQoL, dentists can play an essential role in the awareness of diabetic patients about these problems and improve their quality of life. In addition, we recommend that visiting a dentist be part of the care protocol for diabetic patients.

## Data availability statement

The datasets presented in this article are not readily available because this article is a systematic review and does not have any dataset. Requests to access the datasets should be directed to f_nilchian@dnt.mui.ac.ir.

## Author contributions

YM, KA, ST, MT, and FN researched data, designed the study, reviewed, and edited the manuscript. KA and ST screened, evaluated the quality of the studies, and extract a data spreadsheet. YM checked the data as the third reviewer. Statistical analysis was performed by MT, YM, and FN wrote the first draft of the manuscript. FN is the guarantor of this work and, as such, had full access to all the data in the study and takes responsibility for the integrity of the data, and the accuracy of the data analysis. All authors approved the final version of the manuscript.
