# Supplementary material for: The effect of diabetes mellitus on oral health-related quality of life: A systematic review and meta-analysis study
Source: Front Public Health. 2023 Feb 24;11:1112008. doi: 10.3389/fpubh.2023.1112008 (PMC9998896; doi:10.3389/fpubh.2023.1112008)
Supplement: Supplementary file 1 [file Table_1.DOCX]

Supplementary Material

| No | Database | Search strategy |
| --- | --- | --- |
| 1 | ISI Web of Science | TS=(“Diabetic Patient*” OR Diabet* OR “diabetes mellitus” OR Diabetes OR “Diabetes Mellitus, Type 2” OR “Diabetes Mellitus, Type 1” OR T1DM OR T2DM) AND TS=(“Oral Health Related Quality of Life” OR OHRQoL OR OHRQL) |
| 2 | EMBASE | (‘Diabetic Patient*’:ab,ti OR Diabet*:ab,ti OR ‘diabetes mellitus’:ab,ti OR Diabetes:ab,ti OR ‘Diabetes Mellitus, Type 2’:ab,ti OR ‘Diabetes Mellitus, Type 1’:ab,ti OR T1DM:ab,ti OR T2DM:ab,ti) AND (‘Oral Health Related Quality of Life’:ab,ti OR OHRQoL:ab,ti OR OHRQL:ab,ti) |
| 3 | Scopus | TITLE-ABS-KEY(“Diabetic Patient*” OR Diabet* OR “diabetes mellitus” OR “Diabetes” OR “Diabetes Mellitus, Type 2” OR “Diabetes Mellitus, Type 1” OR T1DM OR T2DM) AND TITLE-ABS-KEY(“Oral Health Related Quality of Life” OR OHRQoL OR OHRQL) |

**Supplementary table 1.** The search strategies of other databases
